# Enhanced Image Processing Using Complex Averaging in Diffusion-Weighted Imaging of the Prostate: The Impact on Image Quality and Lesion Detectability

**DOI:** 10.3390/diagnostics13142325

**Published:** 2023-07-10

**Authors:** Sebastian Werner, Dominik Zinsser, Michael Esser, Dominik Nickel, Konstantin Nikolaou, Ahmed E. Othman

**Affiliations:** 1 Department of Diagnostic and Interventional Radiology, Eberhard Karls University, Tuebingen University Hospital, 72076 Tuebingen, Germany; dominik.zinsser@med.uni-tuebingen.de (D.Z.); michael.esser@med.uni-tuebingen.de (M.E.); konstantin.nikolaou@med.uni-tuebingen.de (K.N.); 2 MR Application Predevelopment, Siemens Healthineers, 91052 Erlangen, Germany; marcel.nickel@siemens-healthineers.com; 3 Department of Neuroradiology, University Medical Center Mainz, 55131 Mainz, Germany; ahmed.e.othman@googlemail.com

**Keywords:** image quality, multiparametric MRI, prostate cancer, diffusion-weighted imaging, high b-value, complex averaging, detection

## Abstract

Diffusion-weighted images of the prostate can suffer from a “hazy” background in low signal-intensity areas. We hypothesize that enhanced image processing (EIP) using complex averaging reduces artifacts, noise, and distortion in conventionally acquired diffusion-weighted images and synthesized high b-value images, thus leading to higher image quality and better detection of potentially malignant lesions. Conventional DWI trace images with a b-value of 1000 s/mm^2^ (b1000), calculated images with a b-value of 2000 s/mm^2^ (cb2000), and ADC maps of 3T multiparametric prostate MRIs in 53 patients (age 68.8 ± 10 years) were retrospectively evaluated. Standard images were compared to images using EIP. In the standard images, 36 lesions were detected in the peripheral zone and 20 in the transition zone. In 13 patients, EIP led to the detection of 8 additional lesions and the upgrading of 6 lesions; 6 of these patients were diagnosed with prostate carcinoma Gleason 7 or 8. EIP improved qualitative ratings for overall image quality and lesion detectability. Artifacts were significantly reduced in the cb2000 images. Quantitative measurements for lesion detectability expressed as an SI ratio were significantly improved. EIP using complex averaging led to image quality improvements in acquired and synthesized DWI, potentially resulting in elevated diagnostic accuracy and management changes.

## 1. Introduction

Prostate cancer is the most common cancer in men. The National Cancer Institute estimates 288,300 new cases of prostate cancer in the US in 2022, i.e., prostate cancer accounts for 14.7% of all new cancer cases, leading to 5.7% of all cancer deaths [1]. Multiparametric MRI (mpMRI) is recommended by the American Urological Association and the European Association of Urology, among others, as an integral part of the initial diagnostic work-up of patients suspected of having prostate cancer [2,3]. Diffusion-weighted imaging (DWI) plays a dominant role in mpMRI since it is the main sequence used in assessing pathology in the peripheral zone of the organ in which 70–75% of cancers originate [4]. Some authors even advocate for bi-parametric MRI using only T2-weighted images and DWI, further elevating the importance of DWI [5]. According to the current version of the Prostate Imaging–Reporting and Data System (PI-RADS^®^, 2019, version 2.1), DWI sequences should include high b-value (HBV) images of at least 1400 s/mm^2^ [4]. The reasoning is that HBV images offer an improved signal contrast between malignant lesions and normal tissue and a lower degree of the T2 shine-through effect compared to lower b-value images, thus resulting in higher sensitivity and specificity compared to standard b-value DWIs [6]. However, drawbacks include additional scanning time, a proneness to artifacts due to longer TEs, a decreased signal-to-noise ratio (SNR), susceptibility effects, and distortions.

One method addressing some of these disadvantages entails using calculated HBV images generated via extrapolation from acquired lower b-value data. Multiple studies suggest that in comparison to acquired HBV images, computed HBV images are non-inferior or even superior concerning the overall image quality; quality of lesion demarcation and detection rate; diagnostic performance; contrast ratios between cancerous and non-cancerous lesions; and suppression of benign tissue, distortion, artifacts, noise, and the contrast-to-noise ratio [7,8,9,10,11]. However, some challenges remain for both computed and acquired HBV images. In DWI, multiple images are acquired and averaged to counter intrinsically low SNR at high b-values. Due to DWI being very sensitive to the diffusion gradients’ phase variations, the magnitude is commonly averaged first, neglecting all phase information. This methodology is very robust but leads to a non-centric noise distribution. The resultant noise bias in the averaged image manifests as a “hazy” background in low signal intensity areas. An adaptive combination of complex-valued images offers a solution, i.e., complex averaging [12]. With this approach, local phase variations between different images are implicitly aligned; therefore, the combined image no longer has a noise bias. The result is an improved image appearance and SNR in low-signal regions [13].

This study aims to qualitatively and quantitatively assess the effects of enhanced image processing (EIP) using complex averaging on conventionally acquired diffusion-weighted images and synthesized high b-value images. We hypothesize that this postprocessing method reduces artifacts, noise, and distortion and thus leads to higher image quality and better detection of potentially malignant lesions.

## 2. Methods and Materials

### 2.1. Population

The institutional review board approved this study and waived the requirement for informed consent. We retrospectively identified consecutive patients who had received multiparametric prostate MRI in our radiology department for four months (March–June 2018). The patients were referred to prostate MRI due to elevated prostate-specific antigen (PSA) levels or suspicious findings at a digital rectal examination or transrectal ultrasound. The medical records were reviewed to determine the most accurate PSA level at the time of the MRI examination. All scans were performed on two 3T scanners (MAGNETOM Skyra or MAGNETOM Prisma^fit^; both Siemens Healthcare, Erlangen, Germany).

### 2.2. Multiparametric MR Protocol and Parameters

The acquisition protocol for diffusion-weighted imaging included the following parameters: three b-values (b = 50, 500, and 1000 s/mm^2^ with 2, 4, and 9 averages, respectively); 4-Scan-Trace diffusion mode; 200 × 200 mm^2^ Field-of-View, 114 × 114 matrix; 30 slices with 3 mm slice thickness; TE = 78 ms; TR = 5700 ms; and TA = 6:42 ms. Additionally, our institution’s standard multiparametric prostate MRI protocol includes axial, sagittal, and coronal T2-weighted images and axial dynamic contrast-enhanced imaging acquired over 2 min.

### 2.3. Enhanced Image Processing (EIP)

In order to improve the resulting image quality, the following steps were prototypically integrated into the scanner reconstruction pipeline. In the first step, all images with the same slice position and diffusion weighting were aligned using non-rigid image registration [14]. Complex-valued images with the same diffusion weighting and direction were then adaptively combined using an algorithm that locally performs a linear phase correction followed by singular-value decomposition and projection on the direction of the largest singular value. The procedure is highly robust to phase variations and keeps the benefits of complex-valued averaging. In more detail, for each pixel $x_{c}$ of a given repetition r, a centered 5 × 5 patch $I_{r}(x)$ is extracted. For each image dimension d, a linear phase coefficient is then determined by $p_{d}=arg\left(\sum_{y}I_{r}^{*}(y+{∆}_{d})I_{r}(y)\right)$, where ${∆}_{d}$ is a unit vector in the direction of d and y runs over all indices for which the argument of the sum can be evaluated. The linear phase correction is then performed by pixel-wise multiplication with $e^{i\sum_{d}p_{d}\;x_{d}}$. In the next step, the spatial indices x are flattened and a singular-value decomposition $I_{r}\left(x\right)=\sum_{s}U_{xs}\sigma_{s}V_{rs}^{*}$ is determined. The projection on the largest singular value is then given by $U_{x_{c}1}\sigma_{1}$ and considered the complex average. The actual implementation of the described algorithm uses sliding window processing and power iterations for the determination of the largest singular-value and eigenvector. After the magnitude extraction and calculation of trace-weighted images, a non-rigid registration was used to align images with different diffusion-weighting [14]. The ADC maps derived from the trace-weighted images show reduced Rician noise bias known from conventional magnitude combinations. Finally, a noise-suppressing extrapolation was used for synthesizing trace-weighted images corresponding to higher diffusion weightings based on a mono-exponential signal model [13]. In the present work, additional images for a diffusion weighting of b = 2000 s/mm^2^ were calculated.

### 2.4. Image Analysis

Image analysis was performed using the trace-weighted images with a b-value of 1000 s/mm^2^ (b1000), the derived ADC maps, and the computed high b-value images with a b-value of 2000 s/mm^2^ (cb2000) as well as the T2-weighted axial images. Two radiologists independently read the images to detect focal lesions in the peripheral and transition zone of the prostate using commercially available software (Centricity Universal Viewer Zero Footprint, GE Healthcare, Chicago, IL, USA). Reading was performed in two separate sessions (4 weeks in between). The readers were blinded to clinical data and the DWI sequence parameters. Examinations were analyzed in a randomized order. The readers identified focal lesions in the peripheral and transition zones and recorded a maximum of one lesion per zone for each patient. The lesion with the higher DWI score was recorded in the case of multiple lesions per zone. The larger lesion was recorded in the case of multiple lesions with the same DWI score. Each detected focal lesion was assigned a DWI score (DWI 1 to 5) using the same criteria as PI-RADS Version 2.1.

Qualitative image analysis included the parameters of image quality, artifacts, and distortion, rated on a 4-point Likert scale (4 = excellent, 3 = good, 2 = moderate, 1 = poor).

Lesion detectability was evaluated qualitatively and quantitatively for the peripheral and transition zones. The qualitative assessment was performed using the 4-point Likert scale described above. Quantitative assessment of lesion detectability was performed in the following manner: we used a software prototype (MR Multiparametric Analysis, syngo.via frontier, Siemens Healthineers). The software allows the copying of regions of interest (ROI) from one image to the exact corresponding location in the respective image of another sequence. A freehand 2D ROI tool was used to measure the signal intensity of the lesions in the ADC map and the b1000 and cb2000 images. We placed one ROI per slice. The number of ROIs depended on the number of slices on which the lesion was identifiable, with one ROI per lesion being the minimum and two ROIs per lesion the maximum number. In case of more than one viable slice, we chose the two consecutive slices on which the lesion was most conspicuous. The ROI area was chosen to be as large as possible, leaving a sufficiently wide margin towards the surrounding parenchyma. The ROI tool delivered the mean signal intensity. Depending on the lesion’s location (peripheral zone or transition zone), two additional circular 2D ROIs were placed in the normal-appearing peripheral zone or transition zone (one left, one right) for reference and objectification purposes. Lesion detectability was defined as the SI ratio between the lesion and the surrounding normal peripheral or transition zone tissue.

### 2.5. Histological and Laboratory Correlation

The medical records were searched for prostate biopsy pathology reports and prostate-specific antigen levels in all patients.

## 3. Statistics

Lesion conspicuity was evaluated separately for lesions in the peripheral and transition zones. The D’Agostino–Pearson test was used to test for the normality of the distribution of quantitative measurements. The Wilcoxon signed-rank test was used to compare qualitative and quantitative measurements with non-normal distribution. A Student’s *t*-test was used to compare qualitative and quantitative measurements with a normal distribution. The interreader agreement was assessed using Cohen’s kappa. A *p*-value < 0.05 was indicative of a significant result. Interrater agreement was evaluated using Cohen’s kappa (κ). Statistical software was used for all analyses (MedCalc Statistical Software version 18.10 (MedCalc Software bv, Ostend, Belgium; http://www.medcalc.org (accessed on 9 June 2023); 2018; GraphPad Prism version 9.0.0 for Windows, GraphPad Software, San Diego, CA, USA).

## 4. Results

### 4.1. Population

**Figure 1:**
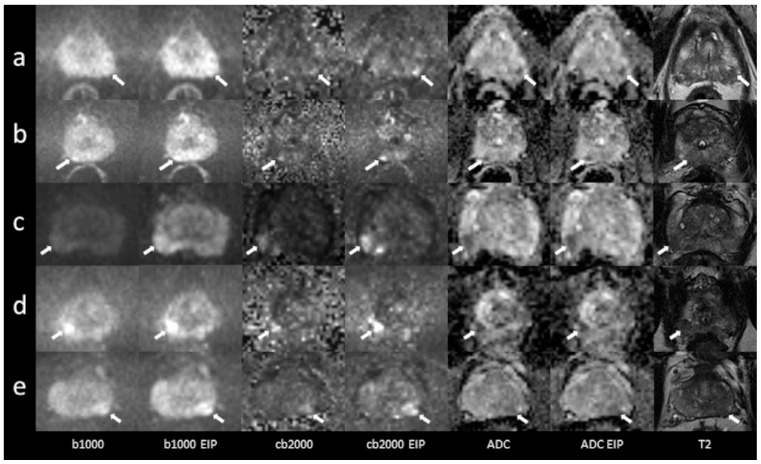
Imaging examples of five patients with peripheral zone lesions: a comparison of standard images and images with enhanced image processing (EIP). (**a**): age, 76 years, PSA, 2 ng/mL. Lesion in the left posterolateral peripheral zone (PZ p) of the apex, measuring 7 mm. Standard DWI score 3, EIP DWI score 4. Histology: adenocarcinoma, Gleason 7a. (**b**): age, 76 years, PSA, 5 ng/mL. Lesion in the right posteromedial peripheral zone (PZ m) of the apex, measuring 5 mm. The lesion was not detected in the standard images. In the EIP images, a DWI score of 4 was assigned. Histology: adenocarcinoma, Gleason 7a. (**c**): age, 59 years, PSA, 5.5 ng/mL. Lesion in the right posterolateral peripheral zone (PZ p) of the base, measuring 12 mm. Standard DWI score 4, EIP DWI score 4. Histology: adenocarcinoma, Gleason 7. (**d**): age, 67 years, PSA, 5.4 ng/mL. Lesion in the right posterolateral peripheral zone (PZ p) of the apex, measuring 9 mm. Standard DWI score 4, EIP DWI score 4. Histology: adenocarcinoma, Gleason 7b. (**e**): age, 56 years, PSA, 6.7 ng/mL. Lesion in the posterolateral peripheral zone (PZ p) of the midportion, measuring 9 mm. Standard DWI score 4, EIP DWI score 4. Histology: adenocarcinoma, Gleason 7. The lesions are denoted by the arrows. b1000: trace-weighted images with a b-value of 1000 s/mm^2^; cb2000: computed high b-value images for a b-value of 2000 s/mm^2^; ADC: ADC map; T2: T2-weighted TSE images; EIP: enhanced image processing.

**Figure 2:**
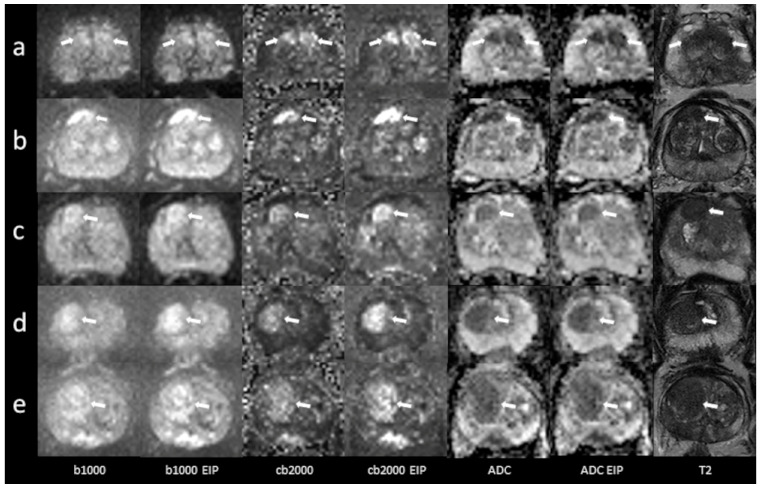
Imaging examples of five patients with transition zone lesions: a comparison of standard images and images with enhanced image processing (EIP). (**a**): age, 78.2 years, PSA, 10 ng/mL. Midline lesion in the anterior transition zone (TZ a) of the midportion, measuring 24 mm. Standard DWI score 5, EIP DWI score 5. Histology: adenocarcinoma, Gleason 6. (**b**): age, 76 years, PSA, 5 ng/mL. Lesion in the right anterior transition zone (TZ a) of the midportion, measuring 17 mm. Standard DWI score 5, EIP DWI score 5. Histology: adenocarcinoma, Gleason 7. (**c**): age, 75 years, PSA, 13.8 ng/mL. Lesion in the right anterior transition zone (TZ a) of the apex, measuring 13 mm. Standard DWI score 4, EIP DWI score 4. Histology: chronic prostatitis. (**d**): age, 85 years, PSA, 7 ng/mL. Lesion in the right anterior and posterior transition zone (TZ a, TZ p) of the apex, measuring 21 mm. Standard DWI score 5, EIP DWI score 5. Histology: chronic prostatitis. (**e**): age, 71 years, PSA, 19 ng/mL. Lesion in the right anterior and posterior transition zone (TZ a, TZ p) of the base and midportion, measuring 29 mm. Standard DWI score 5, EIP DWI score 5. Histology: adenocarcinoma, Gleason 7. The lesions are denoted by the arrows. b1000: trace-weighted images with a b-value of 1000 s/mm^2^; cb2000: computed high b-value images for a b-value of 2000 s/mm^2^; ADC: ADC map; T2: T2-weighted TSE images; EIP: enhanced image processing.

In total, 53 patients were included (mean age 68.8 ± 10 years, range 31–85 years). Forty-three patients underwent MRI on a MAGNETOM Skyra scanner system, while 10 were examined using a MAGNETOM Prisma^fit^. The PSA level at the time of referral to MRI was recorded in all patients (mean 10.1 ± 7.9 ng/mL). In total, 45 patients had undergone a biopsy, which confirmed cancer in 33 patients (Gleason 6, *n* = 4; Gleason 7, *n* = 24; Gleason 8, *n* = 3; Gleason 9, *n* = 2). In five patients, the biopsy diagnosed benign prostate hyperplasia and chronic prostatitis in seven patients. Eight patients did not undergo a biopsy. A detailed report regarding histopathological results, PSA levels, and lesion DWI scores is given in the supplementary table. Imaging examples of 10 patients are presented in Figure 1 and Figure 2.

### 4.2. Image Analysis

#### 4.2.1. Qualitative Analysis of Image Quality

In the b1000 images, the additional processing steps, including the enhanced image processing, resulted in an improved overall image quality rated as 4 compared to a rating of 3 with conventional processing (*p* < 0.001). In the cb2000 images, we found an improvement from 2 to 3 (*p* < 0.001).

The level of artifacts in the cb2000 images received an average rating of 3 with and without additional processing. However, the interquartile range of ratings were smaller with additional processing (IQR 3-3 versus IQR 2-3), resulting in a statistically significant improvement (*p* < 0.001). However, in the b1000 images, artifacts were not significantly reduced (*p* = 0.054).

We found no significant changes in the overall low levels of distortion. They were rated 4 with and without additional processing in the b1000 (*p* = 1.000) and the cb2000 images (*p* = 0.688).

#### 4.2.2. Peripheral Zone

Qualitative lesion detectability Likert scale ratings improved from 3 to 4 via additional processing in the b1000 images (*p* = 0.008). In the cb2000 images, we found an improvement in the ratings’ IQR from 3–4 to 4–4, while the average rating was 4 before and after postprocessing (*p* < 0.001).

**Figure 3:**
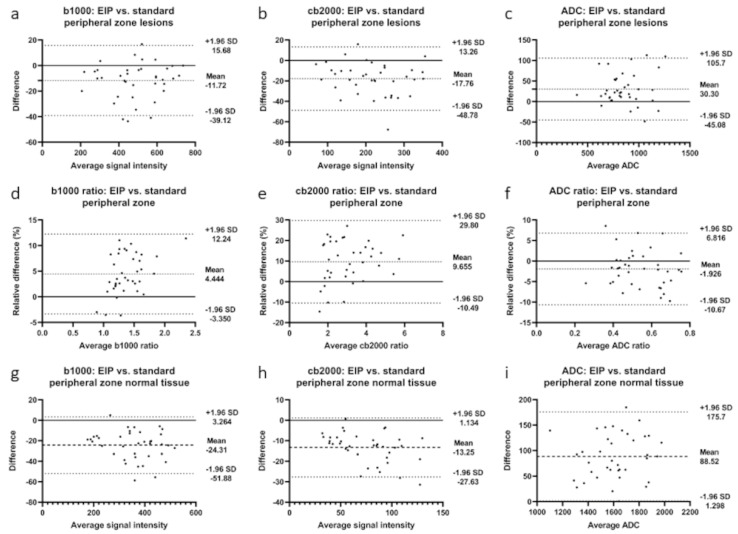
Bland-Altman plots illustrating differences between enhanced image processing and standard images in the peripheral zone for b1000 images, cb2000 images, and ADC maps. (**a**–**c**): differences in lesion signal intensities; (**d**–**f**): differences in signal intensity ratios; (**g**–**i**): differences in normal tissue signal intensities. Mean absolute difference between measurements is indicated by the dashed line. Dotted lines represent the 95% confidence interval.

Correspondingly, quantitative assessment of lesion detectability also showed significant improvement: the median SI ratio increased by 3.8% from 1.31 to 1.36 in b1000 images (*p* < 0.001) and 11.6% from 2.75 to 3.07 in the cb2000 images (*p* < 0.001). The results of the quantitative analysis are presented in Table 1 and Table 2. In addition, Bland–Altman plots depicting the differences between standard and EIP images in lesion signal intensity and ADC values and the differences in SI and ADC ratios are presented in Figure 3.

Regarding lesion classification according to the PI-RADS-based DWI score, EIP led to the detection of two additional lesions (DWI 3 and 4) and both patients were diagnosed with Gleason 7 prostate cancer (Figure 5a,b). Furthermore, postprocessing resulted in upgrading five lesions from DWI 3 to DWI 4 (Figure 6a–e). One of these patients had a Gleason 6 carcinoma and another had a Gleason 7 carcinoma. Using the standard images, we detected 36 lesions with a score of 3 to 5. Of these, 14 lesions (39%) were scored DWI 5, 17 lesions (47%) DWI 4, and 5 lesions (14%) DWI 3. In the EIP images, 38 lesions were detected, of which 14 (37%), 23 (60%), and 1 (3%) of lesions fell into the respective scoring categories.

#### 4.2.3. Transition Zone

In the transition zone, both standard b1000 and processed b1000 images received qualitative lesion detectability Likert scale ratings of 2. The IQR improved from 1–3 to 1–4, albeit without reaching statistical significance (*p* = 0.22). The ratings of the cb2000 images significantly improved from 3 to 4 (*p* = 0.008).

**Figure 4:**
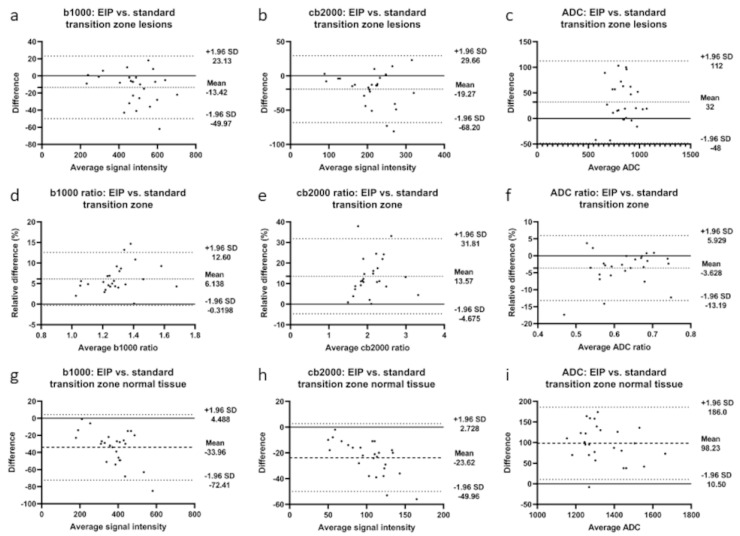
Bland-Altman plots illustrate differences between enhanced image processing and standard images in the transition zone for b1000 images, cb2000 images, and ADC maps. (**a**–**c**): differences in lesion signal intensities; (**d**–**f**): differences in signal intensity ratios; (**g**–**i**): differences in normal tissue signal intensities. Mean absolute difference between measurements is indicated by the dashed line. Dotted lines represent the 95% confidence interval.

Quantitative assessment of lesion detectability showed significant improvement: the median SI ratio increased from 1.23 to 1.31 in b1000 images (*p* < 0.001) and from 1.97 to 2.10 in the cb2000 images (*p* < 0.001). The results of the quantitative analysis are presented in Table 3 and Table 4. In addition, Bland–Altman plots showing the differences between standard and EIP images in lesion signal intensity and ADC values and the differences in SI and ADC ratios are presented in Figure 4.

**Figure 5:**
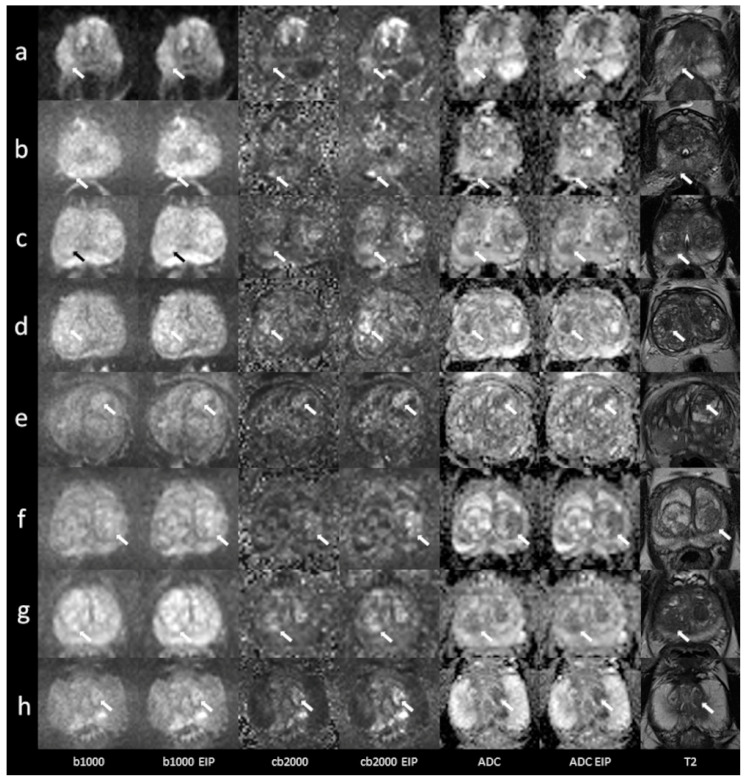
Imaging examples of six patients with peripheral and transition zone lesions were only detected in the images using enhanced image processing (EIP). Comparison between standard images and images with EIP. (**a**): age, 54 years, PSA, 4.6 ng/mL. Lesion in the right posterolateral peripheral zone (PZ p) of the midportion, measuring 9 mm. DWI score 3. Histology: adenocarcinoma, Gleason 7b. (**b**): age, 76 years, PSA, 5 ng/mL. Lesion in the right medial peripheral zone (PZ m) of the apex, measuring 8 mm. DWI score 4. Histology: adenocarcinoma, Gleason 7a. (**c**): age, 69 years, PSA, 7.2 ng/mL. Lesion in the right posterior transition zone (TZ p) of the apex, measuring 12 mm. DWI score 4. Histology: BPH. (**d**): age, 77 years, PSA, 22.6 ng/mL. Lesion in the right posterior transition zone (TZ p) of the midportion, measuring 13 mm. DWI score 4. Histology: chronic prostatitis. (**e**): age, 76 years, PSA, 10 ng/mL. Lesion in the left anterior transition zone (TZ a) of the midportion, measuring 14 mm. DWI score 4. Histology: adenocarcinoma, Gleason 8. (**f**): age, 54 years, PSA, 16.4 ng/mL. Lesion in the left posterior transition zone (TZ p) of the midportion, measuring 15 mm. DWI score 5. Histology: BPH. (**g**): age, 70 years, PSA, 13.3 ng/mL. Lesion in the right posterior transition zone (TZ p) of the apex, measuring 7 mm. DWI score 4. Histology: adenocarcinoma, Gleason 6. (**h**): age, 60 years, PSA, 21 ng/mL. Lesion in the left posterior transition zone (TZ p) of the apex, measuring 13 mm. DWI score 4. Histology: adenocarcinoma, Gleason 7. The lesions are denoted by the arrows. b1000: trace-weighted images with a b-value of 1000 s/mm^2^; cb2000: computed high b-value images for a b-value of 2000 s/mm^2^; ADC: ADC map; T2: T2-weighted TSE images; EIP: enhanced image processing; BPH: benign prostatic hyperplasia.

**Figure 6:**
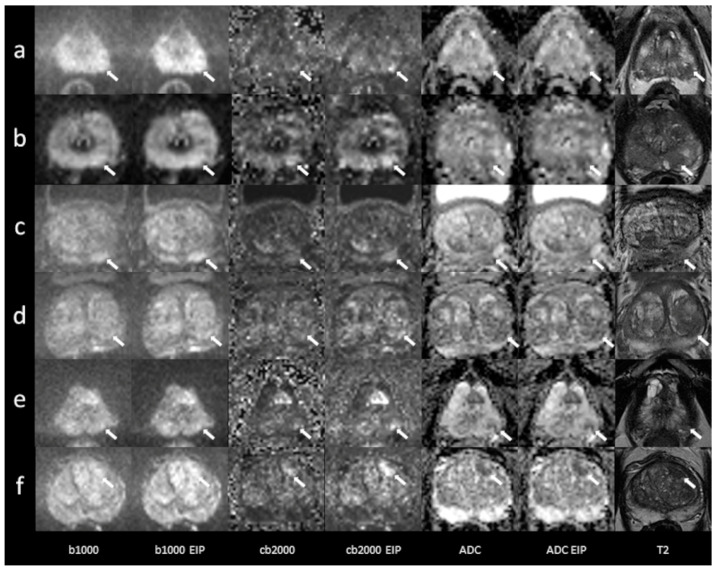
Imaging examples of six patients with peripheral and transition zone lesions were upgraded in the images using enhanced image processing (EIP). Comparison between standard images and images with EIP. (**a**): age, 76 years, PSA, 2 ng/mL. Lesion in the left posterolateral peripheral zone (PZ p) of the apex, measuring 7 mm. Standard images DWI score 3, EIP DWI score 4. Histology: adenocarcinoma, Gleason 7a. (**b**): age, 60 years, PSA, 4.2 ng/mL. Lesion in the left posteromedial peripheral zone (PZ m) of the apex, measuring 5 mm. Standard images DWI score 3, EIP DWI score 4. Histology: not available. (**c**): age, 74 years, PSA, 8.1 ng/mL. Lesion in the left posteromedial peripheral zone (PZ m) of the midportion, measuring 13 mm. Standard images DWI score 3, EIP DWI score 4. Histology: chronic prostatitis. (**d**): age, 54 years, PSA, 16.4 ng/mL. Lesion in the left posterolateral peripheral zone (PZ p) of the midportion, measuring 11 mm. Standard images DWI score 3, EIP DWI score 4. Histology: BPH. (**e**): age, 72 years, PSA, 13 ng/mL. Lesion in the left posterolateral peripheral zone (PZ p) of the apex, measuring 5 mm. Standard images DWI score 3, EIP DWI score 4. Histology: adenocarcinoma, Gleason 6. (**f**): age, 73 years, PSA, 10 ng/mL. Lesion in the left anterior transition zone (TZ a) of the apex, measuring 14 mm. Standard images DWI score 3, EIP DWI score 4. Histology: adenocarcinoma, Gleason 7b. The lesions are denoted by the arrows. b1000: trace-weighted images with a b-value of 1000 s/mm^2^; cb2000: computed high b-value images for a b-value of 2000 s/mm^2^; ADC: ADC map; T2: T2-weighted TSE images; EIP: enhanced image processing; BPH: benign prostatic hyperplasia.

EIP led to the detection of six additional lesions (DWI 4 and 5, Figure 5c–h) and resulted in upgrading one lesion from DWI 3 to DWI 4 (Figure 6f). Of the six patients with additionally detected lesions, three were diagnosed with prostate cancer (Gleason 6, 7, and 8). The patient with the upgraded lesion was diagnosed with a Gleason 7 carcinoma. Using the standard images, we detected 20 lesions with a score of 3 to 5. Of these, 8 lesions (40%) were scored DWI 5, 9 lesions (45%) DWI 4, and 3 lesions (15%) DWI 3. In the EIP images, 26 lesions were detected, of which 9 (34.6%), 15 (57.7%), and 2 (7.7%) lesions fell into the respective scoring categories.

#### 4.2.4. Interrater Agreement

The results of the interrater agreement analysis are presented in Table 5.

## 5. Discussion

The results of our study show that the additional processing steps for enhancing combined trace-weighted images, as well as all derived parameter maps, can improve image quality when applied to acquired b1000 images as well as calculated b2000 images and that artifacts in the calculated HBV images can be significantly reduced. Furthermore, the lesion detectability was ameliorated by postprocessing as assessed by qualitative and quantitative measures in both the acquired and calculated images. Moreover, the complex-averaging method upgraded some lesions from DWI 3 to 4, enabling us to detect additional DWI 3 and 4 lesions.

Our findings are of importance for several reasons. First, fewer artifacts and better image quality probably elevate the reading radiologist’s level of confidence and thus possibly lead to a more accurate diagnosis. A higher detection rate and upgrading lesions can result in a management change. More specifically, upgrading a PI-RADS 3 lesion to a PI-RADS 4 lesion amounts to moving from a situation where a biopsy is debatable to one where it is undoubtedly recommended. The subjectively rated improvement in image quality for the b1000 and cb2000 images was expected. We attribute it to the fact that complex averaging eliminates the noise bias and its manifestation as a hazy background usually seen when magnitude averaging is performed. This is accomplished by combining local image patches without directly phase-correcting the individual images [12]. The better rating for the b1000 images with and without postprocessing compared to the cb2000 images is also not surprising given the innate lower SNR of HBV images and even synthesized ones. In contrast to the cb2000 images, the artifact reduction did not reach statistical significance in the b1000 images. A possible explanation might be that the extrapolation based on an exponential signal model particularly benefits from reduced noise bias in the b = 1000 s/mm^2^ images. We also qualitatively measured levels of distortion, which were very low even without postprocessing and thus did not leave much room for improvement. The qualitative and quantitative improvement of lesion detectability supposedly led to our detection of additional and upgrading some lesions. Unfortunately, the interpretation of these results is somewhat limited because we could correlate these findings with histopathology only in 45 of 53 patients.

A similar study using the proposed method including 84 patients was published by Kordbacheh et al. [13]. In contrast to ours, they only investigated images with high b-values of 2000 s/mm^2^ and compared acquired and calculated images reconstructed using either magnitude or complex averaging. Furthermore, they did not perform a quantitative analysis of image quality or separate analyses for the peripheral and transition zones. Interestingly, in their qualitative analysis, the complex-averaged calculated images received the best rating whereas our highest ratings were given to the acquired lower b-value images with EIP. Nevertheless, both studies found that in the calculated HBV images, the postprocessing subjectively improved the overall image quality. Moreover, similar to our increased detection rate using EIP, they also found increased sensitivity for the complexed-averaged images (73.6% vs. 63.1% in acquired and 68.4% vs. 63.1% in calculated images). Besides implementing the proposed EIP in DWI of the prostate, the technique can also be used in different areas of the body and similar improvements in image quality and lesion detection rates were reported [15,16,17]. Tavakoli et al. have found improved image quality, lesion discernibility, and reduced acquisition times for renal imaging with simultaneous multislice (SMS) DWI with respiratory triggering compared with standard single-shot DWI [17]. Xu et al. have analyzed the technique’s benefits in liver MRI in patients with neuroendocrine tumors and suspected hepatic metastases [16]. They compared SMS DWI with and without motion correction to conventional DWI and achieved superior overall image quality, reduced artifacts, and increased lesion detection rates, especially with motion correction. Moreover, they included a quantitative analysis and demonstrated improved SNR for SMS DWI with and without motion correction compared to conventional DWI. Lastly, Glutig et al. have used the technique in pediatric and young adult patients with cystic fibrosis. They found that SMS DWI with motion correction improves overall image quality and delineation of mesenteric lymph nodes compared to standard DWI with similar SNR and ADC values [15].

Moreover, the proposed method can also be used to reduce scan times, an aspect that we did not analyze in our study. In the studies mentioned above, the authors were able to reduce the scan time by 25% [13], 30% [17], and 32% [15].

Our study has limitations: mainly the limited histopathological correlation owing to the retrospective design. Furthermore, imaging was performed using a pelvic phased-array coil, leaving the effect of our proposed method on images acquired with an endorectal coil unknown. As has been pointed out by Rosenkrantz et al., utilization of an endorectal coil can lead to more pronounced image distortion and susceptibility artifacts and thus might present the potential for synthesized HBV images and complex averaging to deliver an even more significant benefit [10]. On the other hand, recent studies show no relevant diagnostic benefit of endorectal coils and our subjective impression is that their use seems to fall out of favor [18,19]. Finally, regarding the use of calculated instead of acquired HBV images, we recognize that even though they reduce scanning time and provide higher lesion detectability without the drawback of considerably reduced SNR. Nevertheless, they still present a disadvantage: the extrapolated image, which is based on the assumption of Gaussian diffusion behavior, lacks potentially valuable information regarding diffusion hindrance effects at high b-values owing to the non-Gaussian nature of diffusion in the examined tissue. This can result in lower lesion-to-background contrast at high b-values and, in some instances, lower specificity in lesion characterization compared to acquired images [20]. However, in our view, the primary purpose and value of using calculated HBV images lies in providing high lesion detectability at a very low cost and it has been shown that a Gaussian DWI model is adequate for tumor detection [21].

In conclusion, the proposed enhanced image processing method leads to image quality improvements in acquired lower b-values as well as synthesized high b-value images of the prostate, which facilitate a higher detection rate of clinically relevant lesions as well as the upgrading of lesions, potentially resulting in elevated diagnostic accuracy and management changes.

## Figures and Tables

**Table 1:** Ratios of signal intensities and ADC values between lesions and normal prostate tissue in the peripheral zone.

	Median	Relative Difference	Minimum	Maximum	IQR	*p*-Value
b1000 ratio standard	1.31	3.8%	0.90	2.21	0.33	*p* < 0.001
b1000 ratio EIP	1.36		0.88	2.48	0.37	
cb2000 ratio standard	2.75	11.6%	1.60	5.40	1.70	*p* < 0.001
cb2000 ratio EIP	3.07		1.45	6.59	1.84	
ADC ratio standard	0.51	0.0%	0.26	0.77	0.23	*p* = 0.010
ADC ratio EIP	0.51		0.25	0.76	0.20	

**Table 2:** Signal intensities and ADC values in lesions and normal prostate tissue in the peripheral zone.

	Median	Minimum	Maximum	IQR
b1000 standard–lesion	495	217	736	187
b1000 standard–normal	384	186	531	123
cb2000 standard–lesion	229	72	359	119
cb2000 standard–normal	88	35	143	49
ADC standard–lesion	811	387	1204	243
ADC standard–normal	1588	1031	1918	250
b1000 EIP–lesion	480	197	736	190
b1000 EIP–normal	348	167	503	117
cb2000 EIP–lesion	209	68	357	101
cb2000 EIP–normal	70	29	125	42
ADC EIP–lesion	834	403	1314	245
ADC EIP–normal	1655	1171	2034	274

**Table 3:** Ratios of signal intensities and ADC values between lesions and normal prostate tissue in the transition zone.

	Median	Minimum	Maximum	IQR	*p*-Value
b1000 ratio standard	1.23	1.02	1.64	0.09	*p* < 0.001
b1000 ratio EIP	1.31	1.04	1.72	0.17	
cb2000 ratio standard	1.97	1.43	3.24	0.40	*p* < 0.001
cb2000 ratio EIP	2.10	1.50	3.39	0.52	
ADC ratio standard	0.64	0.51	0.79	0.11	*p* < 0.001
ADC ratio EIP	0.62	0.43	0.74	0.12	

**Table 4:** Signal intensities and ADC values in lesions and normal prostate tissue in the transition zone.

	Median	Minimum	Maximum	IQR
b1000 standard–lesion	488	237	714	125
b1000 standard–normal	412	191	623	109
cb2000 standard–lesion	224	87	333	52
cb2000 standard–normal	121	55	193	49
ADC standard–lesion	823	593	1061	156
ADC standard–normal	1249	1099	1628	203
b1000 EIP–lesion	470	228	692	124
b1000 EIP–normal	375	168	538	102
cb2000 EIP–lesion	210	89	327	58
cb2000 EIP–normal	99	43	137	32
ADC EIP–lesion	861	551	1080	151
ADC EIP–normal	1365	1209	1701	175

**Table 5:** Interrater agreement EIP= enhanced image processing.

Item	Cohen’s Kappa
Image quality b1000 standard	0.78
Image quality b1000 EIP	0.80
Image quality cb2000 standard	0.58
Image quality cb2000 EIP	0.66
Artifacts b1000 standard	0.78
Artifacts b1000 EIP	0.74
Artifacts cb2000 standard	0.82
Artifacts cb2000 EIP	0.56
Distortion b1000 standard	0.65
Distortion b1000 EIP	0.57
Distortion cb2000 standard	0.72
Distortion cb2000 EIP standard	0.57
Lesion conspicuity b1000 standard, peripheral/transitional zone	0.85/0.79
Lesion conspicuity b1000 EIP, peripheral/transitional zone	0.88/0.74
Lesion conspicuity cb2000 standard, peripheral/transitional zone	0.88/0.86
Lesion conspicuity cb2000 EIP, peripheral/transitional zone	0.93/0.87

## Data Availability

The data presented in this study are available in the supplementary material (qualitative data.xlsx, quantitative data peripheral zone.xlsx, quantitative data transition zone.xlsx, supplementary table.pdf).

## Supplementary Materials

The following supporting information can be downloaded at: https://www.mdpi.com/article/10.3390/diagnostics13142325/s1.
