# Supplementary material for: Enhanced Image Processing Using Complex Averaging in Diffusion-Weighted Imaging of the Prostate: The Impact on Image Quality and Lesion Detectability
Source: Diagnostics (Basel). 2023 Jul 10;13(14):2325. doi: 10.3390/diagnostics13142325 (PMC10378377; doi:10.3390/diagnostics13142325)
Supplement: Supplementary file 1 [file diagnostics-13-02325-s001.zip › supplementary table.pdf]

Supplementary table Detailed overview showing biopsy results, PSA-level (ng/ml), and DWI score in the peripheral and transition zone for standard images and EIP images. BPH: benign prostate hyperplasia; PSA: prostate-specific antigen; EIP: enhanced image processing

Pathology	Patient number	age	PSA (ng/ml)	Peripheral zone DWI score		Transition zone DWI score	
				standard	EIP	standard	EIP
Gleason 6	14	70.8	13.3				4
Gleason 6	18	78.2	10.0			5	5
Gleason 6	32	72.9	13.0	3	4		
Gleason 6	53	79.9	20.6	5	5	5	5
Gleason 7	2	72.2	9.8			5	5
Gleason 7	5	76.1	2.0	3	4		
Gleason 7	7	67.2	25.0	4	4	4	4
Gleason 7	8	74.2	20.4	5	5		
Gleason 7	9	78.8	6.6	5	5		
Gleason 7	10	76.4	2.0	5	5		
Gleason 7	12	73.3	10.0			3	4
Gleason 7	16	59.0	4.0	5	5		
Gleason 7	19	81.2	8.5	5	5	4	4
Gleason 7	23	78.0	4.9	5	5		
Gleason 7	26	67.8	5.4	4	4		
Gleason 7	30	56.4	6.7	4	4		
Gleason 7	36	61.0	11.2	4	4	4	4
Gleason 7	37	85.7	9.7				
Gleason 7	40	79.9	3.1	4	4		
Gleason 7	41	60.9	21.0	4	4		4
Gleason 7	42	76.6	5.0		4	5	5
Gleason 7	43	68.9	7.3	4	4		
Gleason 7	47	59.1	5.2	4	4		
Gleason 7	48	58.2	5.5	4	4		
Gleason 7	49	53.1	27.6	5	5		
Gleason 7	50	71.8	19.0	5	5	5	5
Gleason 7	51	61.8	5.2	4	4		
Gleason 7	52	54.0	4.6		3	5	5
Gleason 8	4	76.4	23.2	5	5		
Gleason 8	21	67.2	8.3	4	4		
Gleason 8	35	76.5	10.0	4	4		4
Gleason 9	28	55.3	11.0	4	4		
Gleason 9	39	69.4	41.0	5	5		
BPH	1	67.7	11.1	4	4	5	5
BPH	17	69.4	7.2				4
BPH	31	54.4	16.4	3	4		5
BPH	33	71.8	1.0			4	4
BPH	46	71.5	5.4			4	4
chronic prostatitis	3	77.4	22.6				4

chronic prostatitis	15	85.9	7.0			5	5
chronic prostatitis	20	75.5	13.8	4	4	4	4
chronic prostatitis	24	74.0	10.8	4	4		
chronic prostatitis	27	56.5	3.1	4	4		
chronic prostatitis	29	74.3	8.1	3	4	4	4
chronic prostatitis	45	61.6	5.0			3	3
no biopsy	6	65.5	7.2			4	4
no biopsy	11	60.2	4.2	3	4	3	3
no biopsy	13	68.2	4.0			4	4
no biopsy	22	31.2	5.0				
no biopsy	25	78.7	2.6	5	5		
no biopsy	34	65.5	0.9	5	5		
no biopsy	38	78.3	17.1	5	5		
no biopsy	44	58.9	4.3				
